# 14-3-3 theta binding to cell cycle regulatory factors is enhanced by HIV-1 Vpr

**DOI:** 10.1186/1745-6150-3-17

**Published:** 2008-04-29

**Authors:** Diane L Bolton, Robert A Barnitz, Keiko Sakai, Michael J Lenardo

**Affiliations:** 1 Laboratory of Immunology, National Institute of Allergy and Infectious Diseases, National Institutes of Health Bethesda, MD, 20892, USA; 2 ImmunoTechnology Section, Vaccine Research Center, National Institute of Allergy and Infectious Diseases, National Institutes of Health, Bethesda, MD, 20892, USA; 3 Division of Viral Immunology, Center for AIDS Research, Kumamoto University, Kumamoto, Japan

## Abstract

**Background:**

Despite continuing advances in our understanding of AIDS pathogenesis, the mechanism of CD4+ T cell depletion in HIV-1-infected individuals remains unclear. The HIV-1 Vpr accessory protein causes cell death, likely through a mechanism related to its ability to arrest cells in the G_2_,M phase. Recent evidence implicated the scaffold protein, 14-3-3, in Vpr cell cycle blockade.

**Results:**

We found that in human T cells, 14-3-3 plays an active role in mediating Vpr-induced cell cycle arrest and reveal a dramatic increase in the amount of Cdk1, Cdc25C, and CyclinB1 bound to 14-3-3 θ during Vpr_v_-induced G_2_,M arrest. By contrast, a cell-cycle-arrest-dead Vpr mutant failed to augment 14-3-3 θ association with Cdk1 and CyclinB1. Moreover, G_2_,M arrest caused by HIV-1 infection strongly correlated with a disruption in 14-3-3 θ binding to centrosomal proteins, Plk1 and centrin. Finally, Vpr caused elevated levels of CyclinB1, Plk1, and Cdk1 in a complex with the nuclear transport and spindle assembly protein, importin β.

**Conclusion:**

Thus, our data reveal a new facet of Vpr-induced cell cycle arrest involving previously unrecognized abnormal rearrangements of multiprotein assemblies containing key cell cycle regulatory proteins.

**Reviewers:**

This article was reviewed by David Kaplan, Nathaniel R. Landau and Yan Zhou.

## Background

The HIV-1 accessory protein, viral protein R (Vpr), has been shown to be important in viral cytopathicity during virus infection and can kill cells independently when experimentally expressed [1,2]. It is highly conserved, specifically packaged into virions, and facilitates HIV-1 replication in nondividing cells [3], while removal of its homologues in simian immunodeficiency virus results in attenuation of virus replication and disease in rhesus monkeys [4]. The lethal capability of Vpr correlates with its ability to induce cell cycle blockade in G_2_,M [2,5-10]. Although multiple mechanisms have been proposed to explain how Vpr arrests the cell cycle, there is no unifying theory. For example, the DNA damage sensing kinase, ATR, the damaged DNA binding protein 1 component of an E3 ubiquitin ligase, and the scaffolding protein, 14-3-3, have all been shown to mediate Vpr-induced G_2_,M cell cycle arrest [11-17]. Since the majority of these studies were performed in non-lymphocyte human cell lines, a better mechanistic understanding of how Vpr blocks the cell cycle in lymphocytes could help illuminate the mechanism of T cell killing during HIV-1 infection. We thus investigated the role of 14-3-3 in Vpr arrest in T lymphocytes and determined how its function is altered by Vpr and HIV-1 infection.

Progression through the mitotic cell cycle in mammalian cells is controlled by a group of serine/threonine protein kinases termed cyclin-dependent kinases (Cdks) that are activated as a heterodimeric complex with a partner from the family of cyclin proteins. Cdk1-CyclinB is responsible for regulating several events during the G_2_,M transition and progression through mitosis, including chromosome condensation, Golgi network fragmentation, and breakdown of the nuclear lamina. Specific functional substrates include microtubule-binding proteins and proteins involved in translation, ubiquitin-dependent proteolysis, and Cdk1 regulation (e.g. Cdc25 phosphatases). Several negative and positive regulators of both Cdk1 and Cyclins affect kinase activity under normal cycling and cellular stress conditions. For example, Wee1 and Myt1 kinases inhibit Cdk1 activity through phosphorylation of threonine and tyrosine residues, while Cdc25 phosphatases restore activity by dephosphorylation of these residues. Phosphorylation of CyclinB1 is also important for Cdk1-CyclinB activity, as this is required for rapid nuclear entry of CyclinB1 at the end of prophase preceding nuclear envelope breakdown [18]. This gives Cdk1-CyclinB access to key nuclear mitotic substrates.

The 14-3-3 scaffolding proteins have also been implicated in regulation of the G_2_,M phase of the cell cycle. These small, acidic polypeptides of 28–32 kD are highly conserved, expressed in all eukaryotic organisms, and consist of seven different isoforms encoded by distinct genes in mammals with a variety of biological functions. Acting as either hetero- or homodimers, 14-3-3 proteins bind to specific phosphoserine and phosphothreonine sequence motifs and regulate target protein function by compartmental sequestration, altered enzymatic activity, or inhibition/promotion of protein-protein interactions. 14-3-3 regulates the G_2_,M transition by at least two mechanisms: cytoplasmic sequestration of the Cdk1 activating phosphatase, Cdc25C, during most of the natural cell cycle [19,20], as well as cytoplasmic sequestration of Cdk1 itself following DNA damage [21,22]. Recent evidence demonstrated that Vpr- induced G_2_,M arrest is mediated by Vpr binding to 14-3-3, which allows 14-3-3 to bind and inhibit unphosphorylated Cdc25C, preventing Cdk1 activation [11]. As these studies were based on overexpression systems and are conducted in yeast or non-T cell lines, extension of these findings to a more physiological system would help improve our understanding of this important event.

## Methods

### Cell lines and cell cultures

Jurkat T cells were maintained in RPMI complete medium (RPMI 1640 BioWhitaker) supplemented with 10% fetal calf serum (FCS), 100 U/ml penicillin/streptomycin, 2.4 mM L-glutamine, and 50 μM β-mercaptoethanol. All Jurkat experiments were conducted using a CD4^hi ^Jurkat subclone, Jurkat 1.9, generated by single-cell sorting of a low CD4-expressing parental JAK3 cell line [23]. 293T cells were maintained in Dulbecco's modified Eagle's medium (DMEM) supplemented as described above. Adriamycin was added to cultures at 0.2 μg/ml (Sigma) for indicated durations. Cells were synchronized in the cell cycle by treatment with aphidicolin (Sigma; 0.75 μM) for 16 hours, after which cells were released from the block for 10 hours and then subjected to an additional 16 hour block [24].

### Transfection

Transient transfection of Jurkat cells with Vpr expression constructs was performed by electroporation using the Electro Cell Manipulator™ (BTX) apparatus. 4 × 10^6 ^cells resuspended in 0.4 ml of growth medium and 10–15 μg of DNA were electroporated in a 4 mm gap cuvette (Bio-Rad) at 260 V and 1060 microfarads, followed by immediate recovery in growth media for 2–4 days. Transfection efficiency was routinely between 60–80% as determined by flow cytometric analysis of GFP-expressing cells 48 hours post transfection. Typically GFP was co-transfected with other expression plasmids at a ratio of 1:3.3 (e.g. 3 μg GFP with 10 μg pCDNA3-hVpr) as a marker of transfected cells.

### HIV virus stock and infections

HIV viral plasmids were obtained from the NIH AIDS Research & Reference Reagent Repository (ARRRP) unless otherwise indicated. HIV-1 viral stocks of NL4-3_n-GFP _(pNLnGFP-Kp; gift of H. Akari; [25]) were prepared from cell-free supernatant from 293T cells transfected with plasmid using ExGen (Fermentas) according to the manufacturer's recommendations. The *env *gene was mutated to make NL4-3_e- _derivatives for one-round infection purposes by either blunt-end filling of the Nde I site or the Kpn I site in the proviral sequence. *env*- derivatives of NL4-3 were pseudotyped by cotransfection of 293T cells with pLVSV-G at a ratio of 2 μg HIV to 0.5–1 μg pLVSV-G. Viral stocks were harvested 48 hours after transfection, stored at -80°C, and viral titers were assessed by functional MOI determination based on the Poisson distribution (*f*(*x*) = e^-λλx^/*x*! where *x *is a random variable). Using this model, the predicted frequency of 63.2% infected cells is expected after inoculation with a multiplicity of infection (MOI) equal to 1 in a single-round infection. Virion Vpr (Vpr_v_) was delivered to Jurkat cells by either pre-treating target cells with 3TC (10 μM; ARRRP) for 24 hours or using a reverse transcriptase inactive mutant (D186N, RT-; gift of E. Freed, National Cancer Institute, NIH) derivative of NL4-3. Additional viral strains included Vif- [26], Vpr- (deletion mutant of amino acids 22–81), and Vif-Vpr- NL4-3_e-n-GFP_. Typically, Jurkat cells were inoculated at an MOI of 2–5 in 12-well (8 × 10^5 ^cells/well) or 24-well (4 × 10^5 ^cells/well) plates for small-scale infections or in T75 flasks (25 × 10^6 ^cells) for immunoprecipitation and cell fractionation experiments in the presence of polybrene (5 μg/ml; Sigma). Virus was adsorbed for 30 minutes at 37°C, 5% CO_2 _followed by centrifugation for 30 minutes at 800 × *g*. Cultures were maintained at 5–10 × 10^5 ^cells/ml by feeding and splitting cultures as needed.

### Assays for viral production, cell viability, and cell cycle

HIV-1 cytopathicity was assessed by flow cytometric forward and side light scatter or forward scatter and propidium iodide (PI; Sigma) profiles of at least 10,000 live cells daily throughout the course of infection (FACScalibur,^® ^Becton Dickinson). All FACS data analysis was performed using FlowJo software (Tree Star, Inc.). Simultaneously, these samples were measured for HIV-1 provirus expression by GFP fluorescence. Infected viable cell counts were measured by collection for a constant period of time (50 seconds) per sample. To measure DNA content, cells were fixed in 1% paraformaldehyde (in PBS) for 10 minutes, washed once in PBS, resuspended in 70% ethanol while gently mixing, incubated at -20°C for 30 minutes or overnight, washed in PBS, and resuspended in 200 μl DNA staining solution (5 μg/ml PI, 50 μg/ml RNase, 0.45 mg/ml sodium citrate, in PBS) for 30 minutes at 25°C. Alternatively, cells were stained with 10 μM of the cell-permeable DNA dye, DRAQ5 (Axxora, San Diego), for 5 min at 37°C according to the manufacturer's instructions. Stained cells were processed on a FACScalibur^® ^or FACScan (Becton Dickinson) and using the doublet discriminator module (DDM) to exclude cells with DNA signal width greater than the bulk cell population.

### siRNA knockdowns

Jurkat T cells were electroporated as described above with 500 pmol siRNA (Invitrogen Stealth™ RNAi, unless otherwise noted). Target sequences were as follows: 14-3-3 θ 59: UUCUGCUCGAUGCUGAGAUGACCC, 14-3-3 θ 60: UUCGAUCAUCACCACACGCAACUUC, 14-3-3 η 39: UGAUCAGGAACUUGUC

AAGCAGAGA, 14-3-3 β 34: UUAGUGCCAGACCAAGACGAAUUGG, 14-3-3 γ 36: UUCGUUCCUCAUUCGACAGUGGCUC, 14-3-3 ζ 42: AGUAAUUGCAUUAUUA

GCGUGCUGU. Chk1 was targeted using Dharmacon SMARTpool™ siRNA (sequence not provided). Cultures were replenished with fresh media 2 days after transfection and cell density maintained between 5–10 × 10^5^/ml. Cells were infected with HIV 4 days post-transfection and knockdown of protein was assessed by western blot.

### Immunoprecipitation and immunoblotting

For standard western blotting (WB), whole cell protein extracts were prepared by lysis for 30 minutes on ice in modified Laemmli buffer (60 mM Tris, pH 6.8, 10% glycerol, and 2% sodium dodecyl sulfate (SDS)), followed by DNase (Benzonase^® ^nuclease; Novagen) treatment for 30 minutes. The detergent-insoluble fraction was pelleted by centrifugation at 16,000 × *g *in an Eppendorf centrifuge for 10 min at 4°C and supernatants boiled in SDS loading buffer (50 mM Tris-HCl, pH 6.8, 2% SDS, 10% glycerol, 12.5 mM EDTA, 0.02% bromophenol blue) and 20 mM dithiothreitol (DTT). Samples containing equal cell numbers were electrophoresed on a 4–20% Tris/glycine/SDS gel (Novex™) and blotted onto nitrocellulose using a semidry transfer apparatus using the manufacturer's procedure (Biorad).

For immunoprecipitation (IP) experiments, cells were lysed with a mild Nonidet P-40-based IP buffer (0.2% Nonidet P-40, 150 mM NaCl, 10 mM Tris-HCl pH 7.5) for 30 minutes with gentle mixing, normalized for protein concentration quantitated by the BCA™ Protein Assay Kit (Pierce), pre-cleared with Protein G-Agarose (Roche) for 30 minutes, and immunoprecipitated with 3–5 μg antibody for 3 to 6 hours. All steps were performed at 4°C with gentle mixing. Generally, 2–4 mg lysate was used for each IP experiment and an input sample was set aside following the pre-clear step. IP pellets were washed three times in lysis buffer and eluted with SDS loading buffer and separated by SDS polyacrylamide gel electrophoresis (SDS-PAGE) as above.

Cross-linking experiments were performed by treatment of live cells with 1 or 5 mM disuccinimidyl suberate (DSS, Pierce) for 30 minutes at 25°C followed by quenching by addition of 50 mM glycine for 15 minutes at 25°C. Cells were washed in phosphate buffered saline (PBS) twice followed by lysis in IP buffer for 30 minutes at 4°C. Lysates were cleared by centrifugation at 2,000 × *g *for 10 minutes and 120 μg protein was separated on 3–8% Tris-acetate gel (Invitrogen) run in Tris-acetate running buffer (Invitrogen) on ice. Protein was transferred to a nitrocellulose membrane using NuPAGE transfer buffer (Invitrogen) for two hours and blotted as described below.

Cytoplasmic and nuclear separation extracts were prepared by initial lysis in Buffer A (10 mM HEPES, pH 7.9, 10 mM KCl, 0.1 mM EDTA, 0.4% Nonidet P-40, 1.5 mM MgCl_2_, 0.5 mM phenylmethylsulphonylfluoride (PMSF), and 0.5 mM DTT; 0.2 mL/2 × 10^6 ^cells) for 5–10 minutes on ice. Nuclei were pelleted by centrifugation at 750 × *g *for 5 minutes and the cytoplasmic extract (supernatant) was retained. Nuclei were lysed with Buffer C (20 mM HEPES pH 7.9, 420 mM NaCl, 1.5 mM MgCl_2_, 25% glycerol, 0.2 mM EDTA, 0.5 mM PMSF, and 0.5 mM DTT; 20 μl/2 × 10^6 ^cells) for 10–30 minutes on ice mixing as necessary, vortexed vigorously for one minute, and centrifuged at 12,000 × *g *for 10 minutes yielding nucleoplasm in the supernatant. Lysates were separated by SDS-PAGE as above.

Blots were blocked with 2.5% nonfat dry milk in 0.1% PBS-Tween 20 (PBS-T), probed with primary antibody, followed by HRP-conjugated secondary antibody diluted 1:10,000, with three five minute washes in PBS-T after each 30 minute incubation. All antibody probes were performed in 2.5% nonfat dry milk, 0.1% PBS-T. Blots were stripped with Re-Blot Plus Mild (Chemicon International) for 20 minutes and blocked again before reprobing. Bands were imaged with Enhanced Chemiluminescent substrate (Amersham) or SuperSignal^® ^West Dura (Pierce). Complete Protease Inhibitor Cocktail (Roche Diagnostics) was included in all lysis buffers and the Phosphatase Inhibitor Cocktail Set I (Calbiochem) was added to all lysis buffers used in immunoprecipitation experiments. Antibodies used with corresponding catalog numbers are as follows: β-actin (Sigma; A5441), Cdc2 (Santa Cruz; sc-54), Phospho-Tyr15-Cdc2 (Cell Signaling; 9111), Cdc25C (BD Pharmingen; 550922), Cdc25C (Santa Cruz; sc-327), Cdc25C (Calbiochem; CC26; EMSA), Phospho-Ser216-Cdc25C (Cell Signaling; 4901), Centrin (Sigma; C7736), CyclinB1 (Santa Cruz; sc-752 or sc-245), GAPDH (Abcam; ab9484), PARP (BD Transduction Laboratories; P76420), importin β (Sigma; I2534) PKA (Santa Cruz; sc-903), Plk1 (Upstate; 05–844), γ-Tubulin (Sigma; T3559); HIV-1 Vif (NIH ARRRP; 6459), HIV-1 Vpr (gift of K. Strebel, NIAID), 14-3-3 β (Santa Cruz; sc-25276), 14-3-3 θ (Santa Cruz; sc-732), 14-3-3 γ (Santa Cruz; sc-731c), 14-3-3 ζ (Santa Cruz; sc-1019; cross-reacts with β and σ isoforms).

### Centrosome isolation

Centrosomes were isolated as previously described [27] with minor adaptations. Briefly, 50 × 10^6 ^Jurkat T cells were treated with 1 μg/ml cytochalasin D and 0.2 μM nocodazole for 1 hour at 37°C then washed twice in PBS followed by lysis in 5 ml 0.5% NP40 lysis buffer (1 mM Tri1mM Tris-HCl pH 8.0, 0.5% NP40, 0.5 mM MgCl_2_, protease inhibitor, 0.1% 2-mercaptoethanol, phosphatase inhibitor) for 5 minutes on ice. Nuclei and chromatin were pelleted at 2,500 × *g *for 10 minutes and the supernatant was filtered through a 70 μm mesh nylon cell strainer (BD Falcon). PIPES buffer was added to 10 mM and DNase I (Pharmacia) added at 2 units/ml and lysates incubated 30 minutes on ice. 3.5 ml of lysate (~20 mg) was loaded onto a discontinuous sucrose gradient (0.5 ml 70%, 0.3 ml 50%, and 0.3 ml 40% sucrose) in SW55 Ti 5 ml ultracentrifuge tubes (Beckman). All sucrose solutions were prepared w/w in 10 mM PIPES, 0.1% TritonX-100, 0.1% BME. Samples were centrifuged at 120,000 × *g *for 1 h at 4°C. The bottom of the tube was gently pierced with an 18-guage needle and fractions were harvested drop-wise collecting ~200 μl per fraction for 7 fractions. Fractions were diluted into 1 ml 10 mM PIPES and incubated 30 minutes at 4°C with gentle mixing to dissolve the sucrose. Centrosomes were recovered by centrifugation at 16,000 × *g *for 10 minutes.

## Results

### 14-3-3 and Chk1 mediate Vpr G_2_,M cell cycle block in CD4+ T cells

**Figure 1:**
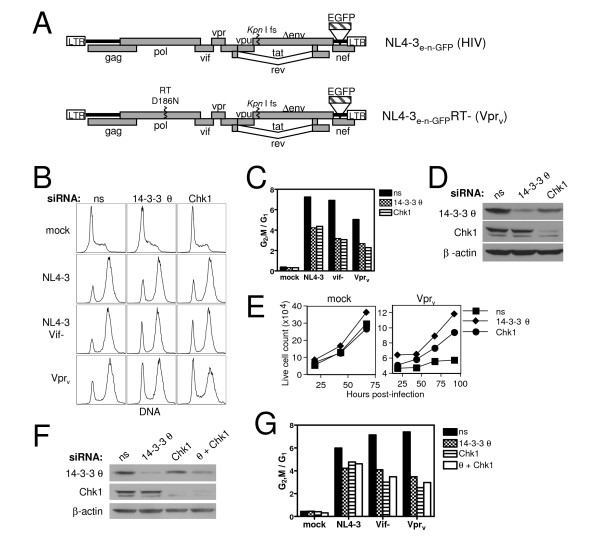
Decreased expression of 14-3-3 θ, β, γ, and Chk1 protects against Vpr_v_- and HIV-1 (*vif*-)-induced cell cycle arrest. (A) Diagram of the basic HIV-1 genomes used throughout the study. *Env*-deficient NL4-3, NL4-3_e-n-GFP_, (top) also lacks *nef*, which was replaced with EGFP. Vpr protein associated with virions (Vpr_v_) was delivered into cells using an RT point mutant, D186N, derivative of NL4-3_e-n-GFP _(bottom). (B) Jurkat T cells were transfected with 500 pmol of nonspecific (ns), 14-3-3 θ, or Chk1 siRNA and four days later mock-infected or infected with either NL4-3_e-n-GFP _(NL4-3), NL4-3_e-n-GFP _Vif- (NL4-3 vif-), or RT- NL4-3_e-n-GFP _(Vpr_v_). Cell cycle arrest by was measured by flow cytometric detection of DRAQ5 DNA staining 44 hours post-infection. DNA analysis of cultures infected with RT+ NL4-3_e-n-GFP _was restricted to actively infected GFP+ cells. Data is representative of six independent experiments. (C) The Dean-Jett-Fox cell cycle model was used for determination of G1 and G_2_,M populations and the ratio is plotted for the FACS data in (B). The transfected siRNA is indicated in the legend. (D) Western blot detection of 14-3-3 θ and Chk1 expression four days post-transfection for the samples in (B) and (C). β-actin was probed as a loading control. (E) Live cell counts were measured for mock-infected and Vpr_v_-infected samples in (B-D) at the indicated times after infection by flow cytometric constant time acquisition. (F) Jurkat cells were transfected as in (B) with the addition of a co-transfected sample that received both 14-3-3 θ and Chk1 siRNA (θ + Chk1). Western blot analysis of 14-3-3 θ, Chk1, and β-actin, as a loading control, is shown. (G) Cell cycle analysis as in (C) for the samples in (F).

**Figure 2:**
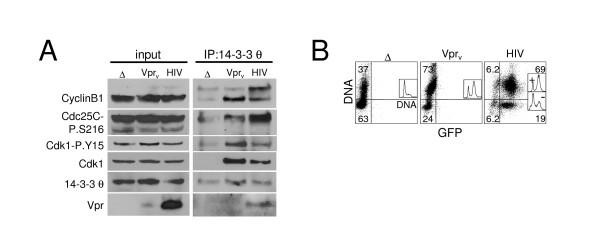
Increased Cdk1, Cdc25C and CyclinB1 association with 14-3-3 θ but stable nucleocytoplasmic distribution during HIV- and Vpr-induced G_2_,M arrest. Jurkat cells were infected as in Fig. 1 with RT- NL4-3_e-n-GFP _virions either with (Vpr_v_) or without (Δ) hVpr supplied in trans or with RT+ NL4-3_e-n-GFP _(HIV; MOI 2). (A) Cell lysates were harvested two days post-infection for immunoprecipitation with 14-3-3 θ and immunoblotting for CyclinB1, Cdc25C-P.S216, Cdc25C, Cdk1-P.Y15, Cdk1, 14-3-3 θ, and Vpr as indicated. The 14-3-3 θ signal in the IP does not reflect poor immunoprecipitation of 14-3-3 θ but rather the result of membrane stripping prior to 14-3-3 θ blotting. (B) DNA content analysis (y-axis) is shown in flow cytometric dot plots against GFP (x-axis) on the right and as a histogram in the inset for the samples in (A). Note that the y-axis of the parent graph becomes the x-axis of the inset graph. The quadrant gate demarcates approximate G1 (lower) and S/G_2_,M (upper) populations and the percentage of cells in each relevant quadrant is indicated. The DNA histogram profile analysis was separated into GFP-positive (+) and negative (-) populations by the x-axis gate for the HIV-infected culture; as expected the infected cells (+) show G_2 _arrest, but the uninfected cells (-) are mostly G1. (C) G_2_,M cell cycle arrest caused by Vpr_v _and HIV infection does not alter the cytoplasmic and nuclear distribution of 14-3-3 θ, Cdc25C, Cdk1, and CyclinB1. Jurkat T cells shown in (A-B) that were infected with NL4-3_e-n-GFP _RT- Δ Vpr (Δ), RT- wt Vpr (Vpr_v_), or NL4-3_e-n-GFP _RT+ (HIV) for two days were lysed and biochemically separated into cytoplasmic and nuclear fractions. Lysate fractions were blotted as in (A) (the lower panel of Vpr blot represents a longer exposure in which Vpr_v _is more apparent), with the addition of probes for HIV-1 Vif, Poly(ADP-ribose) polymerase (PARP) as a nuclear marker, and glyceraldehyde-3-phosphate dehydrogenase (GAPDH) as a cytoplasmic loading control. Cell cycle profiles and GFP expression are shown in (B). (D) Viral lysates (20 μg) of RT- NL4-3_e-n-GFP _virions with (+) or without (-) Vpr were western blotted for CyclinB1, Cdk1, p24, and Vpr as indicated.

The mechanism by which HIV-1 Vpr blocks the cell cycle remains unclear. It was previously shown that the σ and η isoforms of 14-3-3 bind HIV-1 Vpr and play an important role in mediating the G_2_,M cell cycle arrest in the human non-T cell lines, HeLa, HEK 293, and HCT116 [11]. We therefore began our investigation by testing whether siRNA targeting of the various isoforms of 14-3-3 could mitigate Vpr-induced G_2_,M blockade in the CD4+ Jurkat T cell leukemia line. The viral strains used to carry out these studies are shown in Fig. 1A. Since 14-3-3 σ is not expressed in T cells and 14-3-3 θ (or τ) was initially identified as an isoform that uniquely bound active but not inactive Vpr mutants [11,28], we focused on the θ isoform and observed a partial reduction (50–60%) in Vpr-induced G_2_,M arrest upon 14-3-3 θ knockdown as indicated by the decreased ratio of G_2_,M to G_1 _cells (Fig. 1B,C). Nonspecific siRNA, used as a negative control, did not impair the arrest activity. Silencing of Chk1, the DNA damage checkpoint kinase involved in Vpr cell cycle blockade [17], attenuated arrest to the same degree as 14-3-3 θ (Fig. 1B,C). Immunblotting showed a significant reduction of the 14-3-3 θ and Chk1 proteins after RNAi knockdown (Fig. 1D). Similar results were obtained when either virion-delivered Vpr (Vpr_v,_from virus particles that enter cells but cannot establish a productive infection) or endogenously expressed proviral Vpr (NL4-3 Vif-) was used to test Vpr activity. 14-3-3 silencing was generally less potent against proviral Vpr-induced arrest, most likely due to greater Vpr abundance in actively infected cells (data not shown and Fig. 2A). A Vif-deficient strain of NL4-3 was used to assay the activity of Vpr expressed in infected cells because Vif has been shown to independently arrest cells in G_2_,M [26]. However, 14-3-3 θ knockdown did protect against HIV-1-mediated G_2_,M block when both Vif and Vpr were intact, although to a lesser extent than that observed in the absence of Vif. This suggests that Vif likely causes G_2_,M accumulation by a mechanism that does not rely on 14-3-3 θ or Chk1. Thus there are apparently redundant mechanisms for the virus to exert this prominent effect on the cell cycle. We corroborated the G_2_,M arrest alleviation by measuring cell proliferation kinetics (Fig. 1E). While Vpr_v _severely halted cell division in nonspecific siRNA cultures over time, 14-3-3 θ and Chk1 knockdown partially rescued cell expansion, consistent with reduced G_2_,M accumulation in Fig. 1B.

Since 14-3-3 can function as self-dimers or heterodimers with other 14-3-3 isoforms [29-31], and additional isoforms have been shown to bind active Vpr [11], we determined whether other 14-3-3 isoforms play a role in Vpr cell cycle blockade in T cells. Targeting β, η, γ, and ζ isoforms by RNAi revealed that 14-3-3 β and γ knockdown also partially rescued Vpr-induced G_2_,M arrest, although to a lesser degree than 14-3-3 θ knockdown (data not shown). Hence, the θ, β, and γ isoforms of 14-3-3 as well as the checkpoint kinase, Chk1, are important for mediating the G_2_,M arrest caused by Vpr, implicating the DNA damage checkpoint response. Attempts to silence Chk1 and 14-3-3 θ simultaneously impaired knockdown efficiency for 14-3-3 θ (Fig. 1F), but the lack of any further decrease in the G_2_,M/G_1 _ratio relative to silencing of either gene alone suggests that Chk1 and 14-3-3 θ likely mediate Vpr cell cycle block through the same molecular pathway (Fig. 1G).

### Induction of a multiprotein complex containing 14-3-3 θ and G_2_,M regulatory proteins by Vpr and HIV-1 infection

Several cell cycle regulatory functions have been described for 14-3-3 proteins, including cytoplasmic sequestration of the G_2_,M regulatory proteins, Cdc25C and Cdk1 (Cdc2), which limits entry into mitosis [19-22]. To determine if 14-3-3 interactions were altered by Vpr or HIV-1 infection, we examined the association of 14-3-3 θ with Cdk1, CyclinB1, and Cdc25C by co-immunoprecipitation (IP). Vpr_v _significantly increased the amount of Cdk1 and CyclinB1 associated with 14-3-3 θ (Fig. 2A, right panels, IP). Moreover, the Cdk1 associated with 14-3-3 θ included a substantial amount of inactive Cdk1 phosphorylated on tyrosine 15 (P-Y15) (Fig. 2A). Similarly, inactive Cdc25C phosphorylated on serine-216 (P-S216) showed increased association with 14-3-3 θ upon Vpr_v _delivery. Actual HIV-1 infection (RT+) had the same effect, although this result was variable and occasionally less Cdk1 and CyclinB1 were recruited to 14-3-3 θ compared to Vpr_v _treatment (Fig. 2A, HIV lane). This could be due to opposing effects of other HIV-1 proteins that alter the cell cycle, such as Vif or Tat [26,32]. The difference, however, cannot be explained by insufficient Vpr protein since its expression was markedly greater in HIV-infected cells (Fig. 2A, input). Moreover, the G_2_,M blockade was comparable in the Vpr_v _and HIV-infected cultures (Fig. 2B). Note that cells exposed to Vpr_v _are not GFP positive because they do not express a provirus due to an inactive RT and there is not sufficient GFP in the inoculating virions to "donate" fluorescence. Intriguingly, proportionately more high molecular weight CyclinB1, presumably hyperphosphorylated "active" CyclinB1 [18,33], was associated with 14-3-3 θ in HIV-infected samples (Fig. 2A, IP). This upper band was more apparent in the IP than in the whole cell lysate, and thus likely represents a small fraction of total cellular CyclinB1. Thus the virus may drive the active form of CyclinB1 into complex with 14-3-3. This same event could also occur with Vpr_v _but the levels may be too low to detect.

Vpr was also readily observed in the 14-3-3 θ IP (Fig. 2A, HIV) confirming their association [11]. In contrast to HIV infection, Vpr protein delivered by the virion, i.e. Vpr_v_, was generally low or undetectable in the 14-3-3 θ IP (Fig. 2A, IP, Vpr_v_). Thus, the Vpr-14-3-3 θ complex is not obviously stoichiometric with respect to the quantity of Vpr bound to 14-3-3. The augmented levels of Vpr in the complex during HIV infection conditions may reflect Vpr oligomers forming during infection when the protein is more abundant (Fig. 2A, input) [34], as Vpr self-association may be concentration-dependent. In summary, Vpr appears to spark the formation of a multiprotein complex containing 14-3-3 θ, Cdk1, Cdc25C, CyclinB1, and itself. However, the possibility that 14-3-3 θ associates independently with one or more of these proteins rather than as a holo-complex is also a formal possibility.

### Localization of cell cycle regulatory proteins is not altered by Vpr or HIV infection

Although initial work describing 14-3-3 interaction with Vpr suggested that 14-3-3 binding and cytoplasmic sequestration of Cdc25C became more promiscuous in the presence of Vpr [11], we did not observe any gross changes in the cytoplasmic and nuclear distribution of multiple cell cycle proteins, including CyclinB1, Cdk1, Cdk1-P.Y15, Cdc25C-P.S216, and 14-3-3 θ [Additional file 1A]. As expected, inactive Cdc25C-P.S216 was predominantly cytoplasmic, presumably due to 14-3-3 sequestration during normal cell cycling through G1 phase [35]. Total Cdc25C appeared to have the same localization pattern as Cdc25C-P.S216, but possible residual signal from the phospho-probe made these data difficult to interpret (data not shown). The cytoplasmic purity was confirmed by absence of the nuclear protein, Poly(ADP-ribose) polymerase (PARP). Thus, recruitment to 14-3-3 does not alter the nucleocytoplasmic distribution of CyclinB1, Cdk1, or phosphorylated Cdc25C.

Although nuclear levels did not change, Vpr_v_, but not HIV-1 infection, significantly increased cytoplasmic expression specifically of CyclinB1 and Polo-like kinase 1 (Plk1), but not Cdc25C and Cdk1. Plk1, a centrosome-associated mitotic kinase, participates in several mitotic processes including centrosome maturation, Cdc25C activation, inactivation of the Cdk1 inhibitory kinase, Myt1, activation of CyclinB1, and degradation of mitotic Cyclins [36-40]. Furthermore, as we also found in the whole cell lysates in Fig. 2A, the level of Vpr in the cells following virion delivery, Vpr_v, _is markedly less than during HIV-1 infection and barely detectable by western blotting [Additional file 1A]. Nevertheless, Vpr_v _causes G_2_,M blockade as profound as that observed in infected cells (Fig. 2B). Thus, Vpr_v _has additional biological effects on CyclinB1 and Plk1 cytoplasmic protein levels, possibly through regulation of their synthesis, subcellular distribution, or cytoplasmic half-life. It is also possible that the increase in cytoplasmic CyclinB1 and Plk1 reflects protein delivered into cells by HIV-1 virions rather than endogenously produced protein as discussed earlier. To explore this hypothesis, we examined the contents of HIV-1 (NL4-3_RT-_) virions by Western blot analysis. Both CyclinB1 and Cdk1 were present [Additional file 1B], but Plk1 was not detected (data not shown). Moreover, the level of these proteins in the virions was unaltered by the presence of Vpr making it unlikely that virion-delivered protein accounted for the elevation. Rather, the elevated CyclinB1 and Plk1 are most likely derived from endogenous sources in the target cell. Since both CyclinB1 and Plk1 are present in mammalian centrosomes and Vpr causes abnormal centrosome duplication [41-43], these findings could also reflect augmented protein associated with centrosomes. However, the centrosomal marker, centrin, was not similarly increased by Vpr_v _(data not shown).

### Induction of a 14-3-3 θ complex is specific to G_2_,M arresting Vpr

**Figure 3:**
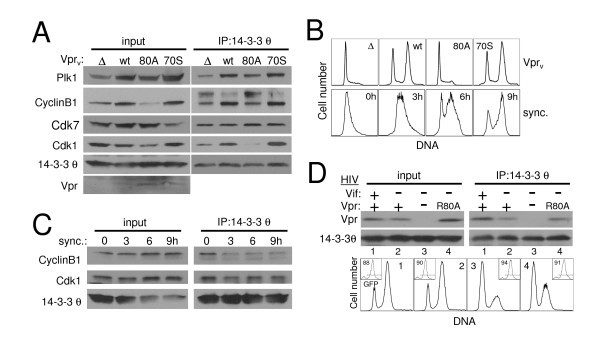
Mutant Vpr fails to stimulate the association of CyclinB1, Cdk1, and Plk1 with 14-3-3 θ but still binds 14-3-3 θ itself. (A) Jurkat cells were infected as in Fig. 2 with RT- NL4-3_e-n-GFP _virions (Vpr_v_) lacking Vpr (Δ) or containing either wild-type (wt), R80A mutant (80A), or I70S mutant (70S) Vpr. Cells lysates were harvested two days post-infection. for immunoprecipitation with 14-3-3 θ antibody (IP: 14-3-3 θ) and the lysates (input) and IP were blotted as indicated with antibodies recognizing Plk1, CyclinB1, Cdk7, Cdk1, 14-3-3 θ, and Vpr. (B) DNA content analysis of the samples in (A), top row (Vpr_v_), and of aphidicolin-synchronized Jurkat cells released from the G1 block for the indicated number of hours (bottom row; sync.). (C) Immunoprecipitation and western blot were performed as in (A) of lysates from the cell cycle synchronized cells shown in (B, bottom row). (D) Jurkat cells were infected with NL4-3_e-n-GFP _(RT+) derivatives containing either wild-type Vpr and Vif (lane 1), Vpr but no Vif (lane 2), neither Vpr nor Vif (lane 3), or R80A mutant Vpr and no Vif (lane 4). Two days post-infection cells were lysed, immunoprecipitated, and immunoblotted as in (A) (top). Flow cytometric DNA content analysis was performed at the time of harvest and shown for the GFP+ (HIV-infected) population of each sample (bottom, numbering corresponds to lane numbers of blots). GFP expression is shown as an inset with the percentage of cells in the GFP-positive gate indicated within the plot.

Since cell cycle perturbations are exerted by Vif, Vpr [26], and possibly other more complicated events during a full HIV infection, we focused on the virion-delivered Vpr_v _system to study the Vpr-specific effect on 14-3-3 binding partners. We first compared Vpr mutants for their ability to drive mitotic proteins into the 14-3-3 θ complex. We found that the well-established R80A point mutant of Vpr, which lacks G_2_,M arrest activity [44], failed to increase Cdk1 and CyclinB1 association with 14-3-3 θ (Fig. 3A, IP; Fig. 3B, top row). Similar results were obtained with the inactive S79A mutant (data not shown). By contrast, both wild-type Vpr and the point mutant, I70S, which does not affect G_2_,M-blocking function [34] (Fig. 3B, top row), increased the complex of Cdk1, CyclinB1, as well as Plk1, with 14-3-3 θ. Western blots showed that similar amounts of mutant and wild-type Vpr protein were delivered (Fig. 3A, input). There was a marked increase in CyclinB1 and Plk1 total protein during G_2_,M arrest by Vpr (Fig. 3A, input, wt and 70S), likely due to enhanced expression of cyclic proteins in pre-mitotic cells, whereas Cdk1 expression was relatively stable. Thus a general increase in protein level could account for augmented 14-3-3 θ association with Cyclin B1 and Plk1 but not Cdk1. To determine if the recruitment was specific, we probed the 14-3-3 θ IP for additional cell cycle regulatory proteins. However, we were unable to detect Chk1, Wee1, or Myt1 (data not shown). Intriguingly, the Cdk1 activating kinase, Cdk7 [45], clearly associated with 14-3-3 θ (Fig. 3A, IP), suggesting that 14-3-3 θ may function as a platform for Cdk1 modification by binding multiple mitotic regulators. Since Cdk7 levels in the IP were unaltered by Vpr_v_-induced G_2_,M arrest, though, the increased recruitment of Cdk1, CyclinB1, and Plk1 to 14-3-3 θ does not extend to all 14-3-3-binding partners. Taken together, these data indicate that induction of Cdk1-14-3-3 θ association depended on the active version of Vpr.

To distinguish whether Vpr induces a high molecular weight (MW) complex(es) or multiple individual complexes containing cell cycle proteins we used the chemical cross-linker disuccinimidyl suberate (DSS) to preserve protein-protein interactions in live cells prior to lysis and SDS-PAGE. The results were complicated. Strikingly, CyclinB1 existed in several higher MW complexes upon wild-type but not inactive R80A Vpr_v _treatment [Additional file 2A, lanes 5 and 6]. Specifically, prominent CyclinB1 bands were detected at approximately 80 kD, 117 kD, 170, and 210 kD [Additional file 2A and 2E]. Of note, these bands were visible but markedly less abundant in cells that did not receive Vpr_v _(lane 4). This suggests that CyclinB1 exists in similar complexes in normal cycling T cells and Vpr simply further induces the high MW weight complexes. While similar analysis of Cdk1 did not indicate any change in its presence within large protein complexes [Additional file 2B], we did observe prominent bands at 117 kD (c), 170 kD (b), and 230 kD (a) in cross-linked cells. The 170 and 230 kD bands correspond to the MW of bands detected on the CyclinB1 blot, and thus may represent CyclinB1-Cdk1-containing complexes. Since the predicted size of a CyclinB1-Cdk1 complex would be approximately 90 kD and therefore smaller than any of the cross-linked species, at least one other component or multiple copies of the proteins must be present within these complexes.

We next attempted to detect 14-3-3 θ, Vpr, and Cdc25C in high MW complexes by western blot, but these experiments were unsuccessful. The chemical cross-linking process may have impaired detection of these proteins by altering or masking epitopes. Nevertheless, these proteins still remain strong candidates for association with CyclinB1 and Cdk1 based on our immunoprecipitation experiments. Similar analyses of cells undergoing HIV-1 infection-induced G_2_,M arrest did not exhibit any increase in CyclinB1 high MW bands [Additional file 2E, lane 6] consistent with its tenuous enhanced association with 14-3-3 during infection (Fig. 2A and 6F). This difference was found even though the infection was robust (71% infected; Additional file 2D) and the extent of the G_2_,M arrest in HIV-infected cells was greater than that caused by Vpr_v _[Additional file 2D, inset histograms]. As a positive control for DSS cross-linking, protein kinase A (PKA) α-catalytic domain was detected as two high molecular weight structures around 80–100 kD upon DSS treatment, consistent with PKA α-catalytic domain heterodimerization with other 40–50 kD catalytic and regulatory domains [46] [Additional file 2F]. Thus, Vpr may cause the formation of high molecular weight complexes with cell cycle regulatory proteins but it is unclear whether these are essential for cell cycle arrest during normal infection.

To determine if 14-3-3 θ binding to cell cycle regulatory proteins is a general characteristic of the G_2_,M phase of the cell cycle or specific to Vpr arrested cells, we examined synchronized Jurkat cells using DNA polymerase inhibition by aphidicolin [47]. Upon release from the drug, most cells were in G_1 _with diploid DNA content (Fig. 3B, bottom row, 0h). By 9 hours, the culture progressed to predominantly G_2_,M phase, characterized by tetraploid DNA, and exhibited a DNA profile similar to cells arrested by Vpr_v _(Fig. 3B, top row, wt). However, co-IP of Cdk1 and CyclinB1 with 14-3-3 θ was not enhanced in these cells (Fig. 3C, IP, 6 and 9 h), despite a slight increase in abundance of these proteins at 6 and 9 hours after release (Fig. 3C, input). Treatment with several chemical inhibitors of mitosis that cause G_2_,M blockade by targeting the cytoskeleton, including demecolcine, nocodazole, and paclitaxel (taxol) also failed to enhance CyclinB1 or Cdk1 association with 14-3-3 θ (data not shown). Thus, the induction of a complex between 14-3-3 θ, CyclinB1, and Cdk1 is specific to Vpr-induced cell cycle blockade.

To confirm that Vpr binding to 14-3-3 θ correlates with its cell cycle arrest activity, we tested this association in infected cells expressing endogenous Vpr because the Vpr interaction with 14-3-3 θ is easier to observe in infected cells than in Vpr_v_-arrested cells (Fig. 2A). Specifically, we mutated Vpr within the physiological context of the full virus HIV-1 NL4-3_e-n-GFP _to the inactive R80A point mutant. These experiments were conducted in a Vif-deficient strain of NL4-3 to eliminate the confounding influence of the G_2_,M arrest activity of Vif [26]. Unexpectedly, R80A mutant Vpr co-immunoprecipitated with 14-3-3 θ to a similar extent as wild-type Vpr (Fig. 3D, top panel), even though Vpr G_2_,M cell cycle blockade was abrogated (Fig. 3D, bottom panel). Thus the reduced function of R80A cannot be explained by its inability to bind 14-3-3 θ. Rather, other components normally present in a 14-3-3 θ complex may be eliminated by this inactivating Vpr mutant. It is also interesting to note that the Vpr co-IP with 14-3-3 θ was weaker in the absence of Vif in HIV-infected cells, suggesting that Vif enhances this interaction. This may partly explain the better association of Vpr to 14-3-3 θ in HIV than in Vpr_v _treated cells (Fig. 2A).

### DNA damage induces a similar 14-3-3 θ complex

**Figure 4:**
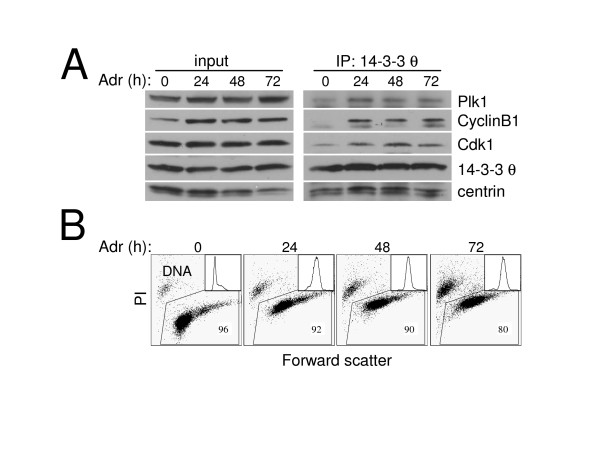
Cell cycle regulatory protein binding to 14-3-3 θ is also enhanced during G_2_,M arrest induced by adriamycin. Jurkat T cells were treated with adriamycin (Adr; 0.2 μg/ml) for 24, 48, or 72 hours (h) or untreated (0) and examined for 14-3-3 θ co-immunoprecipitating (IP) proteins. (A) Western blot analysis of Plk1, CyclinB1, Cdk1, 14-3-3 θ and centrin in whole cell lysates before IP (input) and after 14-3-3 θ IP (IP: 14-3-3 θ) at the time of adriamycin treatment indicated. These data are representative of three independent experiments. Although the amount of 14-3-3 θ appears less in the untreated (0) time point of the IP, this was not a reproducible finding. (B) Viability of the samples used in the IP in (A) determined by flow cytometry detection of propidium iodide (PI) exclusion and large size (high forward scatter). The percentage of viable cells is indicated. Flow cytometric histograms of the DNA content for each sample determined by propidium iodide DNA staining (x-axis) are shown as an inset.

Multiple components of the DNA damage checkpoint are involved in Vpr-induced cell cycle blockade, including Chk1, ATR, and BRCA1 [17,48]. To determine whether elevated 14-3-3 θ binding to Cdk1 and CyclinB1 may be a shared feature of Vpr-induced cell cycle blockage and the mitosis-inhibitory DNA damage response in T lymphocytes, we examined Jurkat cells arrested in G_2_,M by adriamycin [49]. 14-3-3 σ, an isoform not expressed in lymphocytes, binds Cdk1 during G_2_,M arrest caused by adriamycin in the colorectal cancer cell line HCT116 [21], and thus there is precedent for such an interaction. Indeed, the association between 14-3-3 θ and both Cdk1 and CyclinB1 increased dramatically following adriamycin treatment for 24, 48 or 72 hours (Fig. 4A, IP). Plk1 binding to 14-3-3 θ was also enhanced, indicating that mitotic regulatory proteins are specifically elevated in the induced complex. By contrast, the centrosomal resident protein, centrin, was constitutively associated with 14-3-3 θ. Adriamycin powerfully activated the DNA damage checkpoint so that essentially all Jurkat cells accumulated in G_2_,M with 4N DNA content within 24 hours of treatment (Fig. 4B, inset). Furthermore, cell viability decreased from 96% to 80% by 72 hours, indicating emerging toxicity to the DNA damage stimulus (Fig. 4B, dot plot). The magnitude and slow kinetics of adriamycin-induced death are similar to cell death caused by virion-delivered or transfected Vpr, which is usually not apparent until 24–48 hours after initiation of the G_2_,M arrest [2,34,50]. Thus cell cycle dysregulation by both adriamycin and Vpr share several key features: 1) enhanced 14-3-3 θ binding to Cdk1, CyclinB1, and Plk1, 2) profound G_2_,M arrest, and 3) cytotoxicity.

### Centrosomal proteins dissociate from 14-3-3 θ during HIV-induced G_2_,M arrest

**Figure 5:**
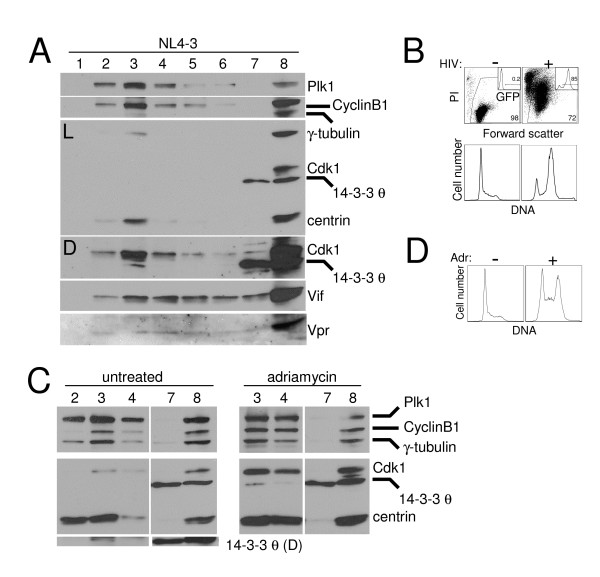
Cell cycle regulatory proteins reside in the centrosome during G_2_,M arrest induced by HIV-1 infection and adriamycin. (A) Western blot analysis of centrosomes isolated from Jurkat cells infected with NL4-3_e-n-GFP _(HIV; MOI of 2) for two days. Centrosomes were isolated by discontinuous sucrose gradient and fractions were collected and separated by SDS-PAGE and western blotted for Plk1, CyclinB1, γ-tubulin, Cdk1, 14-3-3 θ, centrin, Vif, and Vpr. Lanes 1–6 represent fractions from the bottom of the gradient upward, with centrosomes most abundant in lane 3. Whole cell lysates were run in lane 8 and volume from the top of the gradient equivalent to that used for each fraction was run in lane 7 to demonstrate sedimentation of centrosomal proteins through the gradient. "L" indicates a light exposure and "D" indicates a darker exposure of the chemilumigraph of the middle part of the gel. (B) Viability (top) and DNA content analysis (bottom) by flow cytometry for the culture in (A; HIV (+)) and untreated Jurkats without (-) HIV infection. The percentage of viable cells was determined by propidium iodide (PI) exclusion and high forward scatter and is indicated in the lower right corner. Infection efficiency was measured by GFP expression (inset; gated population) and the percentage is indicated. DNA content was measured by flow cytometric detection of DNA stained with propidium iodide. The GFP-positive population was analyzed for the HIV-infected culture. (C) Jurkat T cells were treated with adriamycin (adr; 0.2 μg/ml; right panel) for two days or grown asynchronously (left panel) and centrosomes were isolated by discontinuous sucrose gradients as in (A). Centrosomes were most abundant in lanes 2–3 (untreated cells) or lanes 3–4 (adriamycin treated cells). Lanes were as described in panel A. (D) Cell cycle flow cytometric analysis of Jurkat cells in (C).

Given the growing connection between the centrosome and G_2_,M regulation [42,51,52], we considered the possibility that the altered association between 14-3-3, which localizes to the centrosome [53], and other cell cycle regulatory proteins might reflect disrupted centrosomal localization of these proteins. To test this model, we isolated centrosomes from cytosolic proteins by discontinuous sucrose gradients and determined the abundance of Cdk1, CyclinB1, Plk1, and 14-3-3 θ. Examination of HIV-1-infected G_2_,M arrested cells showed a similar centrosomal protein profile to that of asynchronous and adriamycin-treated cells (Fig. 5A and 5C). Plk1, CyclinB1, and Cdk1 were all highly enriched in the centrosome fraction (lane 3), characterized by the abundance of centrosomal proteins, γ-tubulin and centrin. 14-3-3 θ, by contrast, was primarily found in the cytosol, as it remained in the top of the gradient (lane 7). However, at darker exposures, 14-3-3 θ was also detected in the centrosome fraction in HIV-infected, asynchronous, and adriamycin-arrested cells (Fig. 5A and 5C, "D" exposure, lane 3). These data suggest that 14-3-3 θ does in fact localize to the centrosome, as has been described for other 14-3-3 isoforms [53,54]. Centrosomal location of the θ isoform may be specific to cells in the G_2_,M phase, however, consistent with previous reports of regulated 14-3-3 centrosome localization during cell division [53]. Control experiments showed that the infection was robust (85% of the culture infected) and caused cell cycle arrest and death (Fig. 5B). Similarly, the adriamycin-treated culture accumulated in G_2_,M (Fig. 5D). These data suggest that the general composition of centrosomes in HIV-infected cells does not differ from asynchronous or adriamycin-arrested cells with respect to the cell cycle regulatory proteins Plk1, CyclinB1, Cdk1, or 14-3-3 θ. Vif and Vpr were detected throughout the gradient (lanes 3–7), which suggests that a small fraction of these accessory proteins may localize to the centrosome (Fig. 5A). Thus, enhanced association between 14-3-3 θ and cell cycle proteins during Vpr arrest cannot be explained by failure of these proteins to localize to the centrosomes.

**Figure 6:**
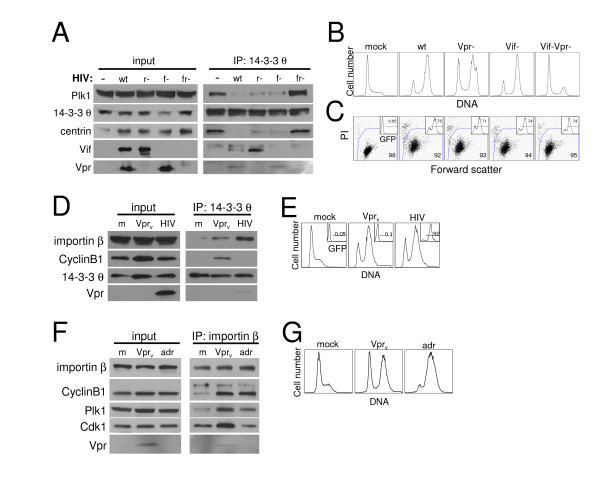
Reduced 14-3-3 θ association with centrosomal proteins, centrin and Plk1, during HIV-1 infection-induced G_2_,M arrest. (A) Jurkat cells were mock-infected (-) or infected with NL4-3_e-n-GFP _derivatives encoding wild-type Vif and Vpr (wt), deleted Vpr (r-), deleted Vif (f-), or double deletion of Vpr and Vif (fr-) at an MOI of 1.5. Lysates were harvested two days post-infection and immunoprecipitated as in Fig. 2 (IP: 14-3-3 θ). Whole cell lysates (input) and IP samples were blotted for Plk1, 14-3-3 θ, centrin, Vif, and Vpr. There appeared to be poor transfer of centrin protein at the left edge of the gel (input lanes). The reduced band intensity for this sample does not reflect decreased centrin abundance as it was well-represented in the IP and similar experiments showed no changes in centrin expression upon HIV-1 infection. (B) DNA content analysis of the samples in (A) by propidium iodide staining. HIV-infected samples were pre-gated on GFP+ cells for DNA analysis. (C) Viability (large plot) and GFP expression by viable cells (inset) for samples in (A) and (B) were measured by flow cytometric detection of propidium iodide (PI) negative, large (high forward scatter) cells and GFP fluorescence (inset histogram), respectively, at the time lysates were harvested. Plots correspond to samples directly above in (B). The gates demarcate viable and GFP-positive cell populations and the percentage of cells within each gate is indicated. (D) Jurkat cells were mock-infected (m), infected with Vpr_v _as in (A), or infected with NL4-3_e-n-GFP _(HIV) and harvested after 40 hours for immunoprecipitation with 14-3-3 θ and immunoblotting with importin β, CyclinB1, 14-3-3 θ, and Vpr. (E) DNA content analysis performed as in (B) is shown for the samples in (D). The inset histogram depicts the percentage of GFP+ cells expressing the NL4-3_e-n-GFP _provirus. The DNA analysis for the HIV-infected sample was performed on the gated GFP population indicated. (F) Jurkat cells were mock-infected (m), infected with RT- NL4-3_e-n-GFP _virions (Vpr_v_) to deliver Vpr protein, or treated with adriamycin (adr). Cell lysates (input) were harvested after 40 hours for immunoprecipitation with importin β (IP) and immunoblotting with importin β, CyclinB1, Plk1, Cdk1, and Vpr as indicated. (G) Cell cycle analysis is shown for the samples in (F) at the time of lysis as measured by propidium iodide DNA staining as in (B).

We therefore considered the possibility that the configuration of 14-3-3 and cell cycle regulatory proteins with centrosomal proteins is altered by the presence of Vpr, and this inhibits normal mitotic activation of Cdk1/CyclinB1 within the centrosome. We tested this hypothesis by co-IP and found markedly decreased centrin associated with 14-3-3 θ upon infection with wild-type HIV-1 (Fig. 6A). Plk1, which also localizes to the centrosome [55], showed a similar striking reduction in 14-3-3 θ association after HIV infection. This contrasted with our findings with Vpr_v _and adriamycin (Figs. 3 and 4). These data indicate that the normal binding of 14-3-3 θ to centrosomal proteins is reduced during HIV-1 infection and that viral components besides Vpr may alter the composition of a 14-3-3 θ complex. To determine if impaired 14-3-3 θ binding to centrin and Plk1 in HIV-1-infected T cells correlated with cell cycle blockade, we examined HIV-1 mutants lacking the G_2_,M arresting proteins, Vpr and Vif. Elimination of either of these genes alone had only modest effects, but the combined mutant virus lacking both Vpr and Vif was severely attenuated in G_2_,M blocking function (Fig. 6B) [26]. While wild-type HIV-1 infection resulted in a massive decrease in 14-3-3 θ-associated centrosomal proteins, infection with *vif*-*vpr*- mutant HIV-1completely restored Plk1 and centrin association with 14-3-3 θ to levels observed in uninfected cells (Fig. 6A). Moreover, *vpr *and *vif *single mutant virus infections, which caused little or no attenuation in G_2_,M arrest activity, respectively, also reduced Plk1 and centrin association with 14-3-3 θ, although less than wild-type virus. Infection efficiencies were matched between the mutant viral strains with 70–75% of the culture expressing the HIV-1 provirus (Fig. 6C, inset) and therefore this difference was not due to inefficient viral replication of the mutant HIV-1 strains. Taken together, these results show that the impaired association between 14-3-3 θ and centrosomal proteins was specific to HIV-1 infection and directly correlated with the extent of the G_2_,M block. Moreover, adriamycin treatment did not cause a similar dissociation between 14-3-3 θ and centrin (Fig. 4A), and thus this effect is not a general feature of G_2_,M arrested cells. We therefore conclude that cell cycle blockade by the Vif and Vpr proteins during HIV-1 infection disrupted protein associations within the centrosome in a manner that correlated with cell cycle blockade.

### Importin β binding to CyclinB1, Cdk1, and 14-3-3 θ is elevated by Vpr

To determine what other cellular proteins bind 14-3-3 θ and therefore might affect the activity of cell cycle proteins associated with the scaffold protein during HIV-1 infection, we performed mass spectrometry on 14-3-3 θ immunoprecipitates from untreated Jurkat whole cell lysates. A matched isotype control antibody was used to distinguish non-specific bands in the immunoprecipitate. Intriguingly, importin β was identified in the 14-3-3 θ-associated fraction (data not shown). To confirm whether 14-3-3 θ and importin β form a complex, we measured importin β in 14-3-3 θ immunoprecipitates and observed low levels in untreated (mock-infected) cells (Fig. 6D, right panel, "m"). The association between these proteins was dramatically increased during HIV-1 infection, however (Fig. 6D), while Vpr_v _caused a milder increase in binding. The G_2_,M arrest by Vpr_v _and HIV-1 was comparable in this experiment (Fig. 6E), and therefore the difference in complex induction cannot be explained by the magnitude of cell cycle block. As in Fig. 2A, the amount of Vpr delivered by Vpr_v _was very low and, in this case, undetectable (Fig. 6D, input), whereas endogenously produced protein in productively infected cells was apparent in the cell lysates and 14-3-3 θ IP. Together, these data suggest that a multi-protein complex consisting of Vpr, 14-3-3 θ, and importin β accumulates in infected cells as well as during Vpr_v _arrest. And since importin β localizes to centrosome-derived spindle poles during the early stages of mitosis [56], it is conceivable that these complexes are derived from centrosomal material.

To test whether CyclinB1 is part of an importin β complex and if Vpr alters the association between them, we immunoprecipitated importin β in cells blocked in G_2_,M by Vpr_v_. Elevated levels of CyclinB1, Plk1, and Cdk1 were bound to importin β compared to untreated cells (Fig. 6F, right panel). In addition, Vpr was present in the importin β IP, consistent with importin β-mediated docking of HIV-1 pre-integration complexes [57]. Since importin β mediates nuclear entry of CyclinB1-Cdk1 [58,59], increased binding among these proteins could simply be a consequence of G_2_,M status, during which the mitotic kinase must be shuttled into the nucleus. However, arrest by adriamycin did not cause increased importin β-Cdk1 association, despite the more pronounced G_2_,M block than that by Vpr_v _and the enhanced association of CyclinB1 and Plk with importin β (Fig. 6G and 6F). The increased binding between Cdk1 and importin β was observed only in cells arrested in G_2_M by Vpr_v_, suggesting that this complex may prevent mitosis possibly by sequestering Cdk1. Therefore, the CyclinB1-importin β complex may be a general characteristic of cells in G_2_,M, while Vpr uniquely induces a Cdk1-importin β complex that could disrupt mitotic progression. Since CyclinB1 is present in both the 14-3-3 θ and importin β IPs, which also co-IP with one another, these proteins likely form a large holo-complex.

## Discussion

Our findings confirm that an association between 14-3-3 and Vpr occurs not only in yeast and transfected 293 cells as observed by Kino et al. [11], but also in HIV-1-infected Jurkat T cells. However, the σ isoform of 14-3-3, which was important for Vpr G_2_,M arrest in 293 cells, is not expressed in lymphoid tissue. We have determined that in T lymphocytes, the θ, β, and γ isoforms are most relevant to Vpr function. Thus 14-3-3 isoforms may perform redundant functions in different cell types depending on the isoforms expressed.

In contrast to the yeast two hybrid and human 293 cell systems [11], we found that Vpr binding to 14-3-3 θ was independent of its ability to block the cell cycle in Jurkat cells. The cell cycle arrest Vpr mutant R80A associated with 14-3-3 θ almost as efficiently as wild-type Vpr in HIV-1 infected cells. Since 14-3-3 isoforms are known to function as homo- and heterodimers, poor interaction between R80A Vpr and 14-3-3 θ in yeast may be due to lack of an additional 14-3-3 isoform that stabilizes R80A binding in mammalian cells. And since 14-3-3 isoforms other than σ were not individually examined in 293 cells by Kino et al., these findings may not reflect Vpr binding interactions with other isoforms or in lymphoid cells which lack 14-3-3 σ. Rather than a 14-3-3 binding defect, we show that cell cycle arrest mutants are impaired for the ability to recruit G_2_,M regulatory proteins to 14-3-3. In addition, this implicates residues other than phosphorylated S79 of Vpr in binding 14-3-3 θ since the R80A mutation prevents S79 phosphorylation [60]. Other 14-3-3 isoforms may bind this residue, though, and be responsible for transporting Cdk1, CyclinB1 in heterodimeric 14-3-3 complexes.

Vpr binding to 14-3-3 has previously been shown to increase cytoplasmic Cdc25C, presumably independent of the Cdc25C phosphoserine residue that usually mediates 14-3-3 binding [11]. However, we were unable to detect any redistribution of phospho-Cdc25C caused by Vpr_v _or HIV-1 infection. Since the prior work was based on overexpression of mutant Cdc25C lacking the phosphorylation site required for 14-3-3 binding, it is possible that Vpr affects only the unphosphorylated Cdc25C population, which was not examined in our studies. Vpr and HIV-1 infection did increase Cdc25C association with 14-3-3, consistent with the idea that Vpr influences 14-3-3-Cdc25C binding. Thus although the distribution of endogenous Cdc25C was not significantly affected by Vpr, altered Cdc25C-14-3-3 binding could impair Cdc25C activation of Cdk1 and prevent normal progression through mitosis.

The prevailing model of Vpr-induced G_2_,M arrest entails inhibition of Cdk1, as evidenced by an increase in the phosphorylated inactive form of the enzyme and impaired kinase activity [5-7]. Despite numerous attempts, we were unable to detect an increase in phosphorylated, inactive Cdk1 in Jurkat cells blocked in G_2_,M by either Vpr_v _or HIV-1 infection. Again, this discrepancy may be due to cell-type specific differences, as previous work on Cdk1 inactivation by Vpr was performed in HeLa epithelial cells. Cell cycle regulation in lymphoid tissue is distinct from that in other mammalian cells during embryogenesis, as hematopoietic cell proliferation defects in Cdk4 and Cdk6 double knockout mice are not exhibited in other cell types [61]. Thus, Cdk1 may play a different role in cell division in epithelial and lymphoid cells. Likewise, a Vpr-induced proliferation block in HeLa cells may be characterized by dramatic inhibition of Cdk1 while other Cdks are more significant in T cells. This is supported by the fact that overexpression of constitutively active Cdk1 blocks Vpr-induced G_2_,M arrest in 293 fibroblasts [5], but not Jurkat cells (data not shown). It is also possible that only a small, critical subset of cellular Cdk1 is inactive, and thus examination of total cell lysates might not reveal this distinct population. In addition, since Jurkat cells are derived from a human T cell leukemia sample that was adapted for continuous growth in culture, its cell cycle regulatory machinery may not be representative of primary T cells. Thus it will be important to confirm these findings in primary cells.

Although we did not observe increased Cdk1 phosphorylation by Vpr or HIV-1 infection, the association of Cdk1 and CyclinB1 with 14-3-3 was significantly altered during Vpr-induced G_2_,M arrest. Similar increases in Cdk1-14-3-3 binding during DNA damage induced G_2_,M arrest are accompanied by cytoplasmic sequestration of Cdk1-CyclinB1 complexes [21]. However, this was not the case in Vpr- or HIV-1-induced G_2_,M arrest. Hence, Vpr increases 14-3-3-Cdk1 binding, presumably as a result of Vpr associating with 14-3-3, but appears to attenuate its activity by a mechanism that does not rely on cytoplasmic sequestration. Since the association of several other G_2_,M regulatory proteins with 14-3-3 also increased, Vpr may nucleate a large multi-protein complex that prevents proper activation of Cdk1. For example, Cdk1 inhibitor kinases, Wee1 or Myt1, could have greater activity on Cdk1 in the complex. This is supported by the apparent increase in phosphorylated, inactive Cdk1 associated with 14-3-3 θ. Furthermore, Wee1 also binds 14-3-3 [62-64], although we were unable to detect Wee1 in 14-3-3 immunoprecipitates (data not shown). The fact that the overall levels of phospho-Cdk1 were unaltered in Vpr-arrested cells could also indicate that the 14-3-3-Cdk1 complex dominantly interferes with unbound Cdk1. In particular, hyperphosphorylated, presumably active CyclinB1, which is required for Cdk1-CyclinB1 nuclear import, was enriched in the 14-3-3 complex in HIV-1-arrested cells, and thus might be unavailable for facilitating nuclear entry of other Cdk1 molecules. Alternatively, a CyclinB1 activating kinase may also be complexed with 14-3-3 and restrained from acting upon CyclinB1 not associated with 14-3-3. Such mechanisms would prevent Cdk1 from entering the nucleus and phosphorylating substrates required for mitotic progression.

It remains unclear why CyclinB1 binding to 14-3-3 θ during HIV-1 infection is variable and occasionally inconsistent with the enhancement induced by Vpr_v_, but other viral proteins may be involved [26,32]. Similarly, Vpr_v _but not HIV-1-infected cell Vpr expression generated high MW CyclinB1/Cdk1 complexes. Thus other HIV-1 proteins may interfere with or disrupt this activity. Alternatively, these complexes may originate from virus-producing cells, as many cell cycle regulatory proteins are packaged into HIV-1 virions. Enrichment of a cell cycle regulatory complex containing CyclinB1 within HIV-1 virions and subsequent delivery of this complex into target cells could substantially increase the amount of higher-order CyclinB1 in target cells. Since these complexes were not consistently amplified in HIV-1-blocked or synchronous G_2_,M cells, they do not appear to be a general feature of G_2_,M-phase cells. We therefore conclude that induction of CyclinB1 multi-protein complexes is specific to Vpr-induced G_2_,M blockade, and this may serve to negatively regulate CyclinB1 activity and maintain the G_2_,M blockade.

The centrosome is emerging as an important cell cycle regulatory organelle where active Cdk1-CyclinB1 is first detected [42]. Moreover, 14-3-3 isoforms ε and γ localize to centrosomes [53], along with several other G_2_,M regulatory proteins. Our data indicate that 14-3-3 θ binding to centrosomal proteins is significantly impaired during HIV-1-induced G_2_,M arrest. This does not appear to reflect poor 14-3-3 localization to centrosomes, but rather a more subtle disruption in the specific binding of 14-3-3 to centrosomal proteins *within *the centrosome. This could have important implications for Cdk1-CyclinB1 activation and nuclear import. For example, binding of the 14-3-3 scaffold protein to centrosomal proteins may form a crucial platform that facilitates Cdk1-CyclinB1 biochemical modification and activation. Increased Cdk1, CyclinB1, or Vpr binding to 14-3-3 could impair the ability of 14-3-3 to interact with centrosomal proteins, which may be required for proper activation of Cdk1 or CyclinB1. Plk1, in particular, phosphorylates CyclinB1, although it is not clear how this affects CyclinB1 activity or how it could be regulated by centrin [42,65]. It is also possible that nuclear import of Cdk1-CyclinB1 occurs via the centrosome, which may serve as a cytoplasmic-nuclear shuttling vessel [52], and the loss of 14-3-3 binding to centrosomal proteins reduces transport efficiency. Although the θ isoform of 14-3-3 had not previously been found to localize to the centrosome [53,54], 14-3-3 θ expression in the centrosome may be unique to certain phases of the cell cycle or to specific cell types. Together, these findings suggest that normal 14-3-3 activity within the centrosome is disturbed in HIV-1 infection-induced G_2_,M arrest and may be responsible for impaired Cdk1 activation. Further evidence that Vpr causes rearrangement, rather than dislocation, of normal protein assemblies is the enhanced association of cell cycle regulatory proteins with importin β, which governs nuclear import and export as well as various functions related to the mitotic spindle.

Vpr is known to cause accumulation of multiple centrosome-like structures and aberrant mitotic spindles [43,66,67]. It is presently not clear how this relates to the reduced 14-3-3-centrosome binding observed in our studies. Abnormal centrosomal duplication during HIV-1 infection apparently requires Vpr [67], whereas HIV-1 lacking Vpr still disrupted 14-3-3 binding to centrosomal proteins, presumably due to Vif. Thus, these two effects on the centrosome are likely unrelated. Since DNA damage-induced G_2_,M arrest increases association between 14-3-3 and the centrosomal kinase, Plk1, HIV-1 arrested cells are distinct from other G_2_,M-arrested cell phenotypes.

Many of the changes in the association between 14-3-3 and cell cycle regulatory proteins in Vpr arrested cells were also observed upon G_2_,M blockade caused by adriamycin-induced DNA damage. This supports the model of Vpr activation of the ATR DNA damage response [17], although it is not clear how this relates to 14-3-3. Consistent with this possibility, our data confirmed that the ATR substrate, Chk1, is important for Vpr-induced G_2_,M arrest. In addition, we provide the first evidence that Chk1 as well as 14-3-3 are of central importance for Vpr cell cycle block in the context of actual HIV-1-infection. Future studies examining the combined effect of Chk1 and 14-3-3 loss will help to determine if these proteins are involved in separate or related mechanisms.

## Conclusion

Although many different mechanisms have been proposed to explain Vpr G_2_,M cell cycle blockade, the data presented herein provide strong support for a 14-3-3-dependent process involving abnormal associations of cell cycle regulatory proteins. Silencing of 14-3-3 θ, in particular, decreased the arrest induced by both virion-associated Vpr and endogenous Vpr during HIV-1 infection in Jurkat T cells. Although 14-3-3 is known to negatively regulate mitosis through cytoplasmic sequestration of Cdk1 and Cdc25C, this mechanism does not appear to be involved in the Vpr arrest. Rather, multiple G_2_,M regulatory proteins accumulate in a complex with 14-3-3 θ, including CyclinB1, Cdk1, and Cdc25C. Similar protein-protein interactions were observed in cells arrested in G_2_,M by DNA damage, but not synchronous G_2_,M cells, suggesting that this process is likely the cause rather than a consequence of G_2_,M status. Intriguingly, the binding of several centrosomal proteins to 14-3-3 θ was dramatically reduced in HIV-1-arrested cells, while importin β increased in association with CyclinB1, Cdk1, and 14-3-3 θ under Vpr arrest conditions. Combined, these data support a model in which mitotic proteins are inactive due to improper post-translational modification conditions in the centrosome or incorporation into a protein complex that prevents access to substrates.

## Competing interests

The authors declare that they have no competing interests.

## Authors' contributions

DLB designed experiments, performed the molecular genetic and biochemistry experiments with RAB, analyzed and interpreted data, generated figures, and wrote the manuscript. RAB performed biochemistry experiments and assisted in designing experiments and editing the manuscript. KS helped design experiments. DLB and MJL conceived of the study. MJL supervised the project, designed experiments and edited the manuscript. All authors read and approved the final manuscript.

## Reviewers' comments

### Reviewer's report 1

David Kaplan, Case Western Reserve University

I have reviewed your manuscript entitled, "14-3-3 theta binding to cell cycle regulatory factor is enhanced by HIV-1 Vpr". My comments follow:

Your manuscript is terrific. You have identified an important area of investigation, the interaction of HIV-1 Vpr with the host cell cycle apparatus, and have made credible progress in your study. Your work has the potential to lead to a greater understanding of HIV-1 pathogenesis, to a therapeutic target for treatment of the infection, and to appreciation for the cellular targets of Vpr. Most importantly, you have assessed the interaction of Vpr with a T cell instead of an epithelial cell. The data presented are convincing. You have combined flow cytometric cell cycle analysis with immunoprecipitation and siRNA inhibition in a clever way. Finally, the manuscript is written in a clear and concise way. In summary, this manuscript represents a significant contribution to the scientific literature.

Nevertheless, I have several suggestions that you may want to consider for revising your manuscript. Specific comments follow:

1. A major feature of this manuscript involves the use of Jurkat cells (which derive from T cells) instead of yeast or transfected 293 cells (which derive from human kidney). Since HIV-1 infects T cells and since cells from different origins express different molecules, there is a powerful rationale for the use of the Jurkat cells. Differential expression of 14-3-3 isoforms emphasizes this choice. Nevertheless, a similar argument could be used in regards to Jurkat cells. Jurkat is derived from a human T cell leukemia sample. It was adapted for continuous growth in culture. It is not the same as primary T cells or nontransformed T cells. In fact, I think it is a misnomer to refer to Jurkat cells as T cells (although I understand that the literature is replete with this usage). I would agree that a study of primary T cells or nontransformed T cell lines or clones should be considered a separate investigation or a continuation of this investigation. However, there is no clear exposition of this point in the manuscript. I find a single sentence that addresses the issue which appears right before the conclusion. This sentence does not indicate that Jurkat cells have been adapted for continuous growth in culture and consequently may have altered the expected patterns of expression of cell cycle proteins. A more extensive consideration of this point would be appropriate in this manuscript.

#### Author's response

We agree that using the Jurkat cell line as a model system for studying regulation of the cell cycle introduces important limitations due to its origins as a leukemia sample adapted for continuous growth in culture. We have elaborated on this point in the conclusions and adjusted the terminology when referring to Jurkat cells as suggested by the reviewer.

2. There is no consideration of statistics in the manuscript. Error bars are not supplied. Consequently, I looked for consistency in the results presented. For instance, in Figure 1C siRNA for 14-3-3 theta clearly decreased the G2 arrest induced by infection with NL4-3 vif-, but in Figure 1F siRNA for 14-3-3 theta induced only a slight change in the G2 arrest induced by infection with NL4-3 vif-.

#### Author's response

The reviewer expresses concern over the level of variation between two siRNA knockdown experiments shown in different panels within Figure 1. These differences result from varying levels in the efficiency of gene knockdown and HIV-1 infection. Since the experiment in Fig. 1F was less representative of multiple replicates, and for reasons described in point #4 below, we removed this panel. As stated in the figure legend, Fig. 1B and 1C are representative of six independent experiments. For example, Fig. 1C and Fig. 1G (formerly Fig. 1I) are highly consistent.

3. In Figure 1D, siRNA for Chk1 appears to have significantly inhibited expression of 14-3-3 theta.

#### Author's response

Although the amount of 14-3-3 θ appears slightly reduced in the Chk1 knockdown in Fig. 1D, this was not observed in multiple repeats of the experiment, including Fig. 1F, and was thus likely a blotting defect.

4. In Figure 1, the targeting of the various isoforms of 14-3-3 by siRNA does not appear to have produce much specificity in effect. For instance, in Figure 1G knockdown of beta has decreased both beta and theta expression. Inhibition of tau has decreased theta expression. Inhibition of gamma appears to have cause a decrease in Chk1. You have indicated that the antibodies to the nu and zeta isoforms crossreact with other isoforms. It may be best to reconsider these data. I am not sure that they add much to the manuscript.

#### Author's response

We agree that the knockdown data of 14-3-3 isoforms in Fig. 1F and 1G are complicated by the possible poor siRNA specificity. We have removed these panels as suggested by the reviewer.

5. I do not understand why there are no GFP positive events in Figure 2B, middle panel, since the Vpr-v vector has GFP.

#### Author's response

Samples treated with Vpr_v _simply receive virion-associated proteins and do not express any genes encoded by the viral genome due to the lack of a functional RT protein. There is insufficient GFP packaged in the viral particle to cause recipient cells to fluoresce. We have added this explanation to the results for clarity.

6. On page 20, you suggest that the monoclonality of the antibody to Chk1 argues against cross-reaction. However, I believe that argument is not correct. Monoclonal antibodies are more prone to cross- reaction than polyclonal antibodies. All antibodies are cross- reactive because the structure of the antibody binding site allows for different structures to be bound. The cross-reactions of different antibodies would not expect to be the same so cross- reaction for polyclonal antibodies is not likely to cause a problem. For monoclonal antibodies cross-reactions can be a problem because all the antibody present has the same cross-reaction.

#### Author's response

We agree with reviewer's point and removed the comment regarding the monoclonal origin of the antibody.

7. In Figure 3D, the flow cytometric panels should be labeled more clearly.

#### Author's response

Due to space constraints, it is not possible to add additional text to label the histograms in Fig. 3D. We clarified in the figure legend that the numbers within each histogram correspond to the lane numbers in the blots directly above.

### Reviewer's report 2

Nathaniel R. Landau, New York University School of Medicine

The Vpr accessory protein of HIV-1 and other lentiviruses has been found to associate with several cellular proteins and it is not yet clear which of these is physiologically relevant.  Bolton et al. show that Vpr can form a complex with 14-3-3, a series of protein scaffolds that directs the cellular localization of cyclin dependent kinases.  Vpr caused 14-3-3 to associate more strongly with Cdk1, Cdc25C and cyclin B1.  In addition, Vpr caused 14-3-3 to bind less well to Plk1 and centrin in the centrosome.  These findings shed light on how Vpr affects the cell cycle.  It will be of interest to understand whether the effects of Vpr on 14-3-3 are caused by the association of Vpr with DDB1 containing E3 ubiquitin ligases or whether this is an independent function for Vpr.  It will also be of interest to understand how these associations contribute to virus replication and pathogenesis.      

There is a lot of good work described in the study. It is well written, thorough and novel. However, there are some weaknesses that need to be addressed. The manuscript is too long. There is a tremendous amount of data presented in the 7 figures, each of which contains several panels. The results, discussion and figure legends, although scholarly, are lengthy. Even the aficionados may not want to read them. The authors should decide on a few panels that can be deleted and a few that can be moved to the Supplement. The results and discussion should be shortened to make a few major points. While the authors have done a huge amount of work, if the paper is too long, its impact will be like a tree falling in the forest. There needs to be a clear take-home message conveyed by the manuscript.   

#### Author's response

The reviewer suggests shortening the length of the Results and Discussion sections to better focus the manuscript. We have eliminated a few figure panels and moved some to the supplemental material. We have also edited the text of the Results and Discussion to be more concise.     

Secondly, there were recently a set of important papers published on Vpr that show its interaction with a DDB1 containing E3 ubiquitin ligase. The authors make no mention of this work. This may leave the impression that the authors are somewhat disconnected from the rest of the Vpr field. The authors may want to at least mention this work.   

#### Author's response

We have added a comment to the introduction mentioning the recent published body of work on Vpr binding to DDB1 and its relevance to the cell cycle block activity of Vpr.     

Thirdly, the authors portray the early work on Vpr that shows its role in nuclear import as established fact. It is not. The authors may want to soften their statements about this model for Vpr function, as it is not generally accepted in the field.   

#### Author's response

As suggested by the reviewer, we have softened the tone of any statements regarding the role of Vpr in mediating import of the virus into the nucleus.

### Reviewers' report 3

Yan Zhou, Johns Hopkins University School of Medicine

In "14-3-3 θ binding to cell cycle regulatory factors is enhanced by HIV-1 Vpr", Bolton et al. investigate the role of scaffold protein 14-3-3 in Vpr-mediated cell cycle arrest in Jurkat T cells. A previous study using the yeast two-hybrid system and non-lymphocyte cell lines suggested that Vpr directly binds to 14-3-3 and facilitates the cytoplasmic sequestration of Cdc25C. The siRNA knockdown experiment in this study convincingly demonstrates that endogenous 14-3-3, particularly 14-3-3 θ, plays an important role in Vpr-mediated cell cycle arrest.

The authors performed immunoprecipitation experiments to probe the components of 14-3-3 θ complex formed upon Vpr-induced cell cycle arrest. In addition to single-round viral infection, the authors took the elegant approach of introducing virion associated Vpr (Vpr_v_) into cells with an RT-minus virus to focus on Vpr-mediated cell cycle arrest. Control experiments included mock infected cells, cells synchronized by aphidicolin, cells arrested at G_2,_M by adriamycin, and cells infected with virus carrying an inactive mutant Vpr. The authors demonstrated that enhanced binding of cell cycle regulators to 14-3-3 θ is characteristic of cells arrested at G_2,_M, although the association of several components with 14-3-3 θ varied among cells arrested by Vpr_v_, HIV-1 infection, and adriamycin. In case of Vpr_v_, enhanced binding of cdk1, Cdc25C, and CyclinB1 to 14-3-3 θ was observed.

The direct binding of Vpr to 14-3-3 θ would suggest an initiating role for the multi-protein complex in Vpr-mediated cell cycle arrest. The authors could not prove this in cells arrested by Vpr_v_. Instead, they found that both active and inactive Vpr bound to 14-3-3 θ in infected cells. Therefore, it remains possible that the complex executes rather than initiates Vpr-induced G_2,_M arrest.

The authors demonstrated that the formation of the multi-protein 14-3-3 θ complex correlates with G_2,_M arrest. However, previously reported cytoplasmic sequestration of Cdk1 and Cdc25C as well as hyperphosphorylation of Cdk1 was not observed in this system, probably due to cell type specific regulation of cell cycle. In an effort to explore the mechanism underlying cell cycle arrest, the authors demonstrated that the 14-3-3 θ complex localizes to centrosomes in HIV-1 infected cells and cells treated with adriamycin. Interestingly, the binding of 14-3-3 θ to centrosomal proteins, centrin and Plk1, is disrupted in HIV-1 infection but not in adriamycin treated cells. It will be informative to test if impaired association between centrin and 14-3-3 θ also occurs in cells treated with Vpr_v_. The authors further demonstrated enhanced binding of the nuclear transport and spindle assembly protein importin θ to the 14-3-3 θ complex in cells arrested by Vpr_v _and HIV-1 infection. In light of these results, the authors proposed that abnormal assembly of 14-3-3 θ complex affects the activity of cell cycle regulators or their access to substrates.

This study has revealed the important role of 14-3-3 θ in orchestrated events in Vpr-induced G_2,_M arrest. Recent studies suggested the involvement of damaged DNA binding protein 1 and Cul4A in Vpr-induced cell cycle arrest. It will be interesting to determine the spatial and temporal relationship between ubitquitin-ligase complex, Vpr, and the 14-3-3 θ complex described in this study. It will also be important, as the authors point out at the end of this manuscript, to confirm all of these findings in primary CD4^+ ^T cells.

## Supplementary Material

Additional file 1 Biochemical analysis of cell cycle protein subcellular distribution. The data represent the nucleocytoplasmic distribution of Cdk1, Cdc25C and CyclinB1 during HIV- and Vpr-induced G_2_,M arrest. (A) Jurkat cells shown in Fig. 2A–B that were infected with NL4-3_e-n-GFP _RT- Δ Vpr (Δ), RT- wt Vpr (Vpr_v_), or NL4-3_e-n-GFP _RT+ (HIV) for two days were lysed and biochemically separated into cytoplasmic and nuclear fractions. Lysate fractions were blotted as in Fig. 2A (the lower panel of Vpr blot represents a longer exposure in which Vpr_v _is more apparent), with the addition of probes for HIV-1 Vif, Poly(ADP-ribose) polymerase (PARP) as a nuclear marker, and glyceraldehyde-3-phosphate dehydrogenase (GAPDH) as a cytoplasmic loading control. Cell cycle profiles and GFP expression are shown in Fig. 2B. (B) Viral lysates (20 μg) of RT- NL4-3_e-n-GFP _virions with (+) or without (-) Vpr were western blotted for CyclinB1, Cdk1, p24, and Vpr as indicated. Click here for file

Additional file 2 Biochemical analysis of cell cycle proteins in chemically cross-linked cells. The data depict CyclinB1 accumulation in high molecular weight complexes following Vpr_v _G_2_,M arrest. (A-C) Jurkat cells treated with RT- NL4-3_e-n-GFP _to deliver wild-type (lane 2, "w"), R80A (lane 3, "A") or no (lane 1, Δ) Vpr for two days were cross-linked with disuccinimidyl suberate (DSS, 1 mM). Whole cell lysates were separated by 3–8% Tris-Acetate SDS-PAGE and transferred to membranes that were blotted for CyclinB1 (A; 55 kD) and Cdk1 (B; 34 kD). High molecular weight bands of interest are indicated with, "a," "b," "c," and "d." (C) Flow cytometric histograms of the DNA content analysis for samples in (A-B). (D-F) Jurkat cells mock-infected (lane 1, "m") or infected with RT- (Vpr_v_; lane 2, "V") or RT+ (HIV; lane 3, "H") NL4-3_e-n-GFP _for two days were analyzed as in (A-C) for DNA content (D) and higher-order CyclinB1 complex formation by DSS cross-linking (E). Propidium iodide DNA staining is plotted against GFP, and the inset histograms depict the DNA content for the entire culture or the GFP positive (+) and GFP negative (-) subsets for the HIV-infected culture. Note that the y-axis of the parent graph is represented by the x-axis in the inset graph. The percentage of cells in the GFP+ gate is indicated. (E) Western blot analysis of untreated control (-) and DSS cross-linked (+) cells for CyclinB1 (top) and Cdk1 (bottom). No higher molecular weight bands were seen in the Cdk1 blot due to membrane stripping following the CyclinB1 blot. The same membrane was stripped and reprobed for the α-catalytic subunit of PKA (F; 40 kD) as a positive crosslinking control. Click here for file
