# Supplementary material for: 14-3-3 theta binding to cell cycle regulatory factors is enhanced by HIV-1 Vpr
Source: Biol Direct. 2008 Apr 29;3:17. doi: 10.1186/1745-6150-3-17 (PMC2390528; doi:10.1186/1745-6150-3-17)
Supplement: Additional file 1 — Biochemical analysis of cell cycle protein subcellular distribution. The data represent the nucleocytoplasmic distribution of Cdk1, Cdc25C and CyclinB1 during HIV- and Vpr-induced G2,M arrest. (A) Jurkat cells shown in Fig. 2A–B that were infected with NL4-3e-n-GFP RT- Δ Vpr (Δ), RT- wt Vpr (Vprv), or NL4-3e-n-GFP RT+ (HIV) for two days were lysed and biochemically separated into cytoplasmic and nuclear fractions. Lysate fractions were blotted as in Fig. 2A (the lower panel of Vpr blot represents a longer exposure in which Vprv is more apparent), with the addition of probes for HIV-1 Vif, Poly(ADP-ribose) polymerase (PARP) as a nuclear marker, and glyceraldehyde-3-phosphate dehydrogenase (GAPDH) as a cytoplasmic loading control. Cell cycle profiles and GFP expression are shown in Fig. 2B. (B) Viral lysates (20 μg) of RT- NL4-3e-n-GFP virions with (+) or without (-) Vpr were western blotted for CyclinB1, Cdk1, p24, and Vpr as indicated. [file 1745-6150-3-17-S1.ppt]

## Slide 1
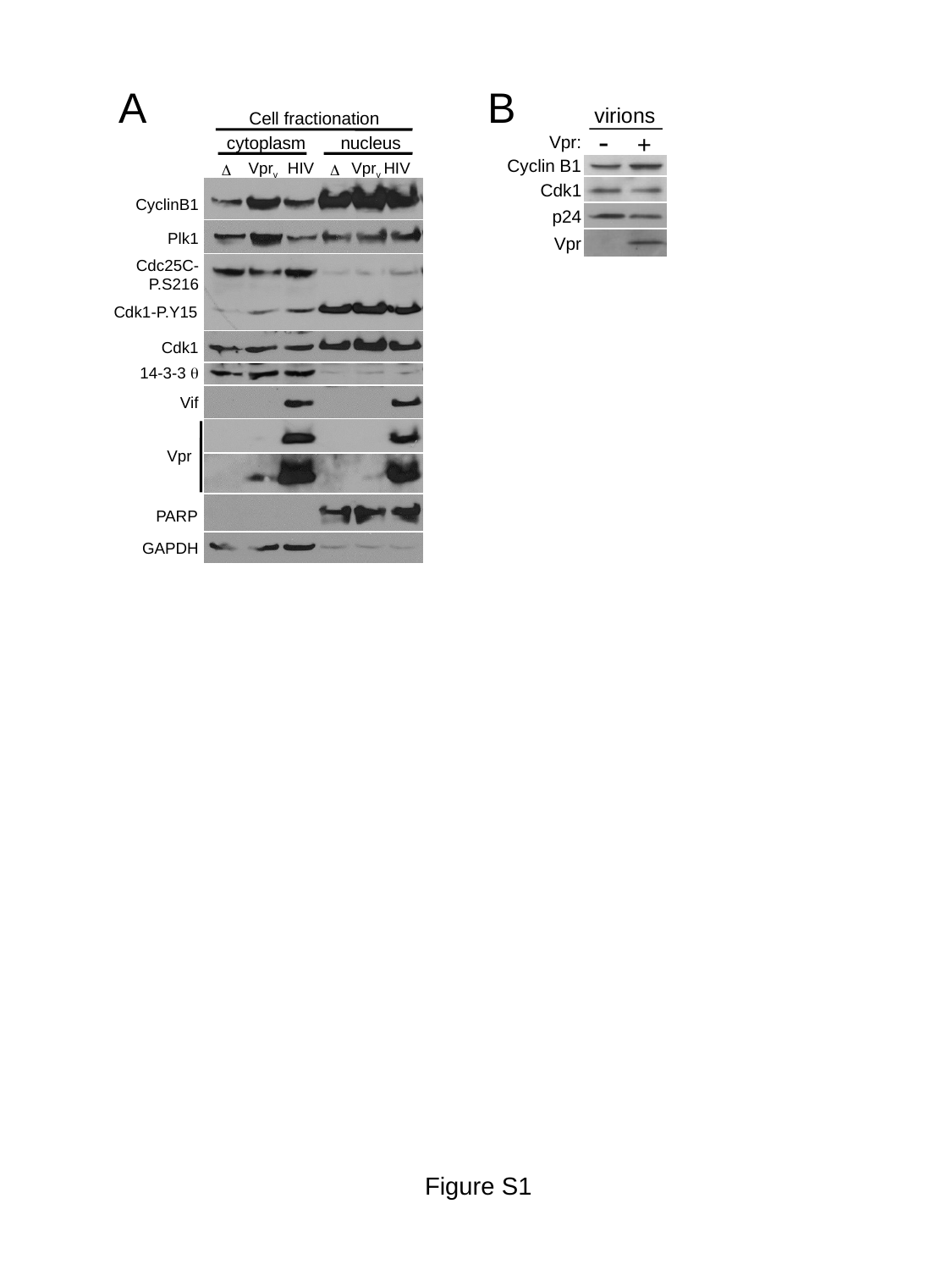

A
B
virions
Cell fractionation
-
+
Vpr:
cytoplasm
nucleus
Cyclin B1
Vprv
HIV
Vprv
HIV
Δ
Δ
Cdk1
CyclinB1
p24
Plk1
Vpr
Cdc25C-
P.S216
Cdk1-P.Y15
Cdk1
14-3-3 
Vif
Vpr
PARP
GAPDH
Figure S1
