# Supplementary material for: 14-3-3 theta binding to cell cycle regulatory factors is enhanced by HIV-1 Vpr
Source: Biol Direct. 2008 Apr 29;3:17. doi: 10.1186/1745-6150-3-17 (PMC2390528; doi:10.1186/1745-6150-3-17)
Supplement: Additional file 2 — Biochemical analysis of cell cycle proteins in chemically cross-linked cells. The data depict CyclinB1 accumulation in high molecular weight complexes following Vprv G2,M arrest. (A-C) Jurkat cells treated with RT- NL4-3e-n-GFP to deliver wild-type (lane 2, "w"), R80A (lane 3, "A") or no (lane 1, Δ) Vpr for two days were cross-linked with disuccinimidyl suberate (DSS, 1 mM). Whole cell lysates were separated by 3–8% Tris-Acetate SDS-PAGE and transferred to membranes that were blotted for CyclinB1 (A; 55 kD) and Cdk1 (B; 34 kD). High molecular weight bands of interest are indicated with, "a," "b," "c," and "d." (C) Flow cytometric histograms of the DNA content analysis for samples in (A-B). (D-F) Jurkat cells mock-infected (lane 1, "m") or infected with RT- (Vprv; lane 2, "V") or RT+ (HIV; lane 3, "H") NL4-3e-n-GFP for two days were analyzed as in (A-C) for DNA content (D) and higher-order CyclinB1 complex formation by DSS cross-linking (E). Propidium iodide DNA staining is plotted against GFP, and the inset histograms depict the DNA content for the entire culture or the GFP positive (+) and GFP negative (-) subsets for the HIV-infected culture. Note that the y-axis of the parent graph is represented by the x-axis in the inset graph. The percentage of cells in the GFP+ gate is indicated. (E) Western blot analysis of untreated control (-) and DSS cross-linked (+) cells for CyclinB1 (top) and Cdk1 (bottom). No higher molecular weight bands were seen in the Cdk1 blot due to membrane stripping following the CyclinB1 blot. The same membrane was stripped and reprobed for the α-catalytic subunit of PKA (F; 40 kD) as a positive crosslinking control. [file 1745-6150-3-17-S2.ppt]

## Slide 1
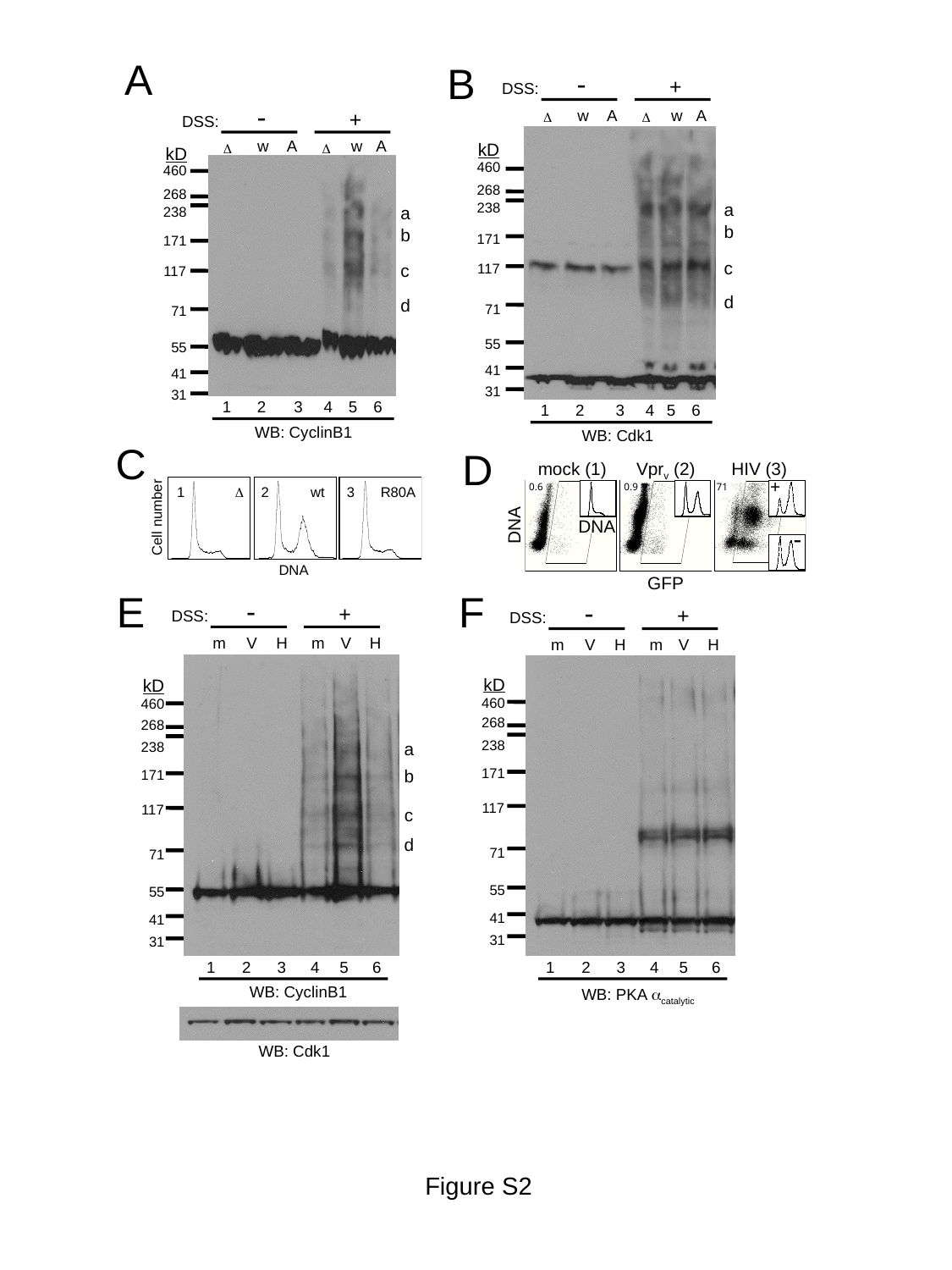

A
B
-
+
DSS:
-
+
w
A
w
A
Δ
Δ
DSS:
w
A
w
A
kD
Δ
Δ
kD
460
460
268
268
a
238
a
238
b
b
171
171
c
c
117
117
d
d
71
71
55
55
41
41
31
31
1
2
3
4
5
6
1
2
3
4
5
6
WB: CyclinB1
WB: Cdk1
C
D
mock (1)
Vprv (2)
HIV (3)
+
1
Δ
2
wt
3
R80A
Cell number
DNA
DNA
-
DNA
GFP
E
F
-
-
+
+
DSS:
DSS:
m
V
H
m
V
H
m
V
H
m
V
H
kD
kD
460
460
268
268
238
a
238
171
b
171
117
117
c
d
71
71
55
55
41
41
31
31
1
2
3
4
5
6
1
2
3
4
5
6
WB: CyclinB1
WB: PKA αcatalytic
WB: Cdk1
Figure S2
